# Combined therapy of somatostatin analogues with pegvisomant for the treatment of acromegaly: a meta-analysis of prospective studies

**DOI:** 10.1186/s12902-020-0545-2

**Published:** 2020-08-18

**Authors:** Lingyun Ma, Daohuang Luo, Ting Yang, Songtao Wu, Min Li, Chaoyang Chen, Shuang Zhou, Lingyue Ma, Ye Wu, Ying Zhou, Yimin Cui

**Affiliations:** 1 Department of Pharmacy, Peking University First Hospital, No.6, Da Hong Luo Chang Street, Beijing, 100034 China; 2 College of Pharmacy, Peking University Health Science Centre, Beijing, China; 3 Department of Pediatrics, Peking University First Hospital, Beijing, China

**Keywords:** Somatostatin analogues, Pegvisomant, Acromegaly, Meta-analysis

## Abstract

**Background:**

Acromegaly is a rare, chronic and severe disease. Drug therapy including somatostatin analogues (SAs), dopamine receptor agonists and growth hormone receptor antagonists (pegvisomant, PEG) are commonly used to treat patients who do not respond to surgery. The use of combination therapy with PEG and SAs has become more common over the last decade. We performed this study to accurately evaluate the effect of combination therapy of SAs with PEG on acromegalic patients.

**Methods:**

*PubMed, EMBASE, The Cochrane Library, ClinicalTrials.gov, Scopus, Web of Science, Chinese Biomedical Literature Database and Trip database* were searched for relevant studies. Prospective clinical trials treating acromegaly with the co-administration of SAs and PEG were included. We performed a meta-analysis by using *Stata 12.1*. Sensitivity analysis was conducted to explore heterogeneity.

**Results:**

Nine studies were included in this meta-analysis. The overall rate of serum insulin-like growth factor 1 (IGF-1) normalization was 66% (95% CI: 52–78%; I^2^ = 62.59%). The combination therapy did not significantly change patients’ fasting plasma glucose (ES: 0.011 mmol*L^− 1^; 95% CI: − 0.374 to 0.397 mmol*L^− 1^; *P* = 0.954) or glycosylated haemoglobin (ES: − 0.074%; 95% CI: − 0.166 to 0.315%; *P* = 0.544) while decreasing the fasting plasma insulin (ES: − 21.487 pmol*L^-1^; 95% CI: − 35.713 to − 7.260 pmol*L^-1^; *P* = 0.003). Elevation of liver enzyme levels was found in 14% (95% CI: 8 to 21%) of the patients. There was no significant difference for serious adverse events and treatment discontinuation due to adverse event between SAs monotherapy group and combination therapy group.

**Conclusions:**

Combined therapy of SAs and PEG effectively normalized IGF-1 levels in most of the patients whose IGF-1 level was greater than the upper limit of normal after high dose SAs monotherapy. The therapy also decreased significantly FPI levels with a neutral effect on glucose parameters in acromegaly patients. Moreover, elevated liver enzyme levels were observed in a small number of patients, which suggests a need for liver function monitoring.

**Trial registration:**

We have our protocol registered in PROSPERO. (Registration number: CRD42019115549).

## Background

Acromegaly is a rare, chronic and severe disease in which growth hormone (GH) is overproduced mainly due to GH-secreting pituitary. GH circulates and stimulates production of insulin-like growth factor 1 (IGF-1) from the liver [1]. Therefore, serum levels of IGF-1 are usually used for assessment of disease control. Moreover, some acromegalic patients are bothered with impaired glucose tolerance and type 2 diabetes mellitus, with a prevalence of the latter ranging from 19 to 56% [2]. The mechanism is not completely understood, but it may be related to insulin resistance due to overproduction of GH [3].

Surgery is recommended as the first-line treatment option when an experienced surgeon is available and the tumour is resectable [4]. However, not all patients can be cured by surgery. Moreover, surgery of microadenomas has a remission rate of more than 85% [5, 6]. Thus, other strategies such as drug therapy are necessary in some acromegalic patients.

Currently, available medical treatments include somatostatin analogues (SAs: octreotide, lanreotide or pasireotide), dopamine receptor agonists and growth hormone receptor antagonists (pegvisomant (PEG)). SAs, which suppress GH secretion by binding to somatostatin receptors, remain the primary medical treatment option if surgery is not possible or curative [7]. The IGF-1 normalization rate of SAs monotherapy varies from 17 to 69% [8–12]. Different from SAs, PEG inhibits production of IGF-1 by binding to GH receptors and preventing their dimerization [13]. PEG monotherapy has been shown to be effective for normalization of IGF-1 in about 70% of cases [14, 15]. As for the safety issue, an elevation of liver transaminase levels is the main adverse drug reaction in the patients treated with SAs or PEG [14]. There are two well-known types of hepatic enzyme disturbance: hepatocellular and cholestatic. Cholestatic disturbances are most often related to treatment with somatostatin analogues, hepatocellular to treatment with PEG [16]. In patients with unsatisfactory results after SAs monotherapy, PEG as monotherapy or in association with SAs is generally the next treatment attempt [7]. Recently, an analysis of the ACROSTUDY demonstrated that the use of combination therapy with PEG and SAs has become more common over the last decade [17].

A previous meta-analysis investigated glucose metabolism during the combination therapy [18]. However, the authors did not study other important efficacy such as IGF-1 or safety profile of the treatment. Therefore, as an update, we performed this meta-analysis to get an accurate and full picture of the effect of the combined SAs and PEG therapy on acromegalic individuals based on all published reports.

## Methods

This meta-analysis was reported according to PRISMA statement.

### Search strategy

We searched for studies in *PubMed, EMBASE, The Cochrane Library, ClinicalTrials.gov, Scopus, Web of Science, Chinese Biomedical Literature Database and Trip database (up to February 2020).* There were no limits on publication date. The following words were used to build the search strategies: acromegaly, octreotide, lanreotide, somatostatin, pasireotide and pegvisomant (see the full search strategy in Additional file 1).

### Inclusion and exclusion criteria

Studies were included if they met the following eligibility criteria: (1) prospective trials; (2) patients diagnosed with acromegaly; (3) patients using an SAs in association with PEG; (4) the length of study was at least 3 months; and (5) studies reporting efficacy outcomes (IGF-1 normalization rate after the combination therapy) or glucose metabolism outcomes before and after the treatment or safety outcomes (the number or percentage of patients with elevated liver enzyme levels after the treatment, number or percentage of patients discontinuing treatment due to adverse events and number or percentage of patients with serious adverse event). We excluded reviews, retrospective studies, animal studies and publications not in English or Chinese.

Firstly, the identification of potentially relevant studies, by reviewing titles and abstracts, was completed by two authors. Then, the full texts of the remaining studies were reviewed by two authors independently to identify the final studies for meta-analysis. We solved any disagreements with open discussion.

### Data extraction and quality assessment

Data were extracted from the included studies by two reviewers. If data were not reported, we contacted the corresponding authors to obtain necessary data. The following data were extracted: (1) study design; (2) baseline characteristics (number of patients, age, sex, previous treatment, the length of study, mean or median dosage of SAs and PEG); (3) number or percentage of patients with normalized age- and sex-adjusted IGF-I levels; (4) fasting plasma glucose (FPG), fasting plasma insulin (FPI), and glycosylated hemoglobin (HbA1c), before and after combination treatment; (5) number or percentage of patients with elevated liver enzyme levels; (6) number or percentage of patients discontinuing treatment due to adverse events in the combination therapy group and SAs monotherapy group; and (7) number or percentage of patients with serious adverse event in the combination therapy group and SAs monotherapy group. Only the last follow-up assessment was considered for all outcomes.

Quality assessment was performed by the investigators using the Cochrane risk-of-bias algorithm for controlled trials [19] and modified Methodological Index for Non-randomized Studies [20] for non-comparative studies.

GRADE was applied to assessed the evidence quality of two safety outcomes (serious adverse event and treatment discontinuation due to adverse event).

### Statistical analysis

Meta-analysis was performed with *Stata 12.1.* Heterogeneity was evaluated via Chi-square and I^2^ statistical tests [21]. If the I^2^ of heterogeneity< 50%, we will chose fixed-effects model, otherwise, the random-effects model will be chosen. Meta-analysis of event rates (normalization rate of IGF-1 level, elevation rate of liver enzyme level) were conducted using double arcsine transformation. The rates are presented with lower and upper limits of 95% confidence intervals (CIs). The overall effect size of the other two safety outcomes (number or percentage of patients discontinuing treatment due to adverse events and number or percentage of patients with serious adverse event) was presented as odds ratio (OR) and 95%CI. We computed the mean difference between post-values and pre-values of other data (FPG, FPI, and HbA1c) and get summary statistics for the overall difference. The estimated effect size (ES) was reported as the mean difference and 95% CIs.

To assess sensitivity, when the effect size was dependent on one or two trials (e.g. a large trial), these trials were dropped from the analysis. Statistical significance was assumed when *P*-values were less than 0.05.

## Results

### Literature screen

**Fig. 1:**
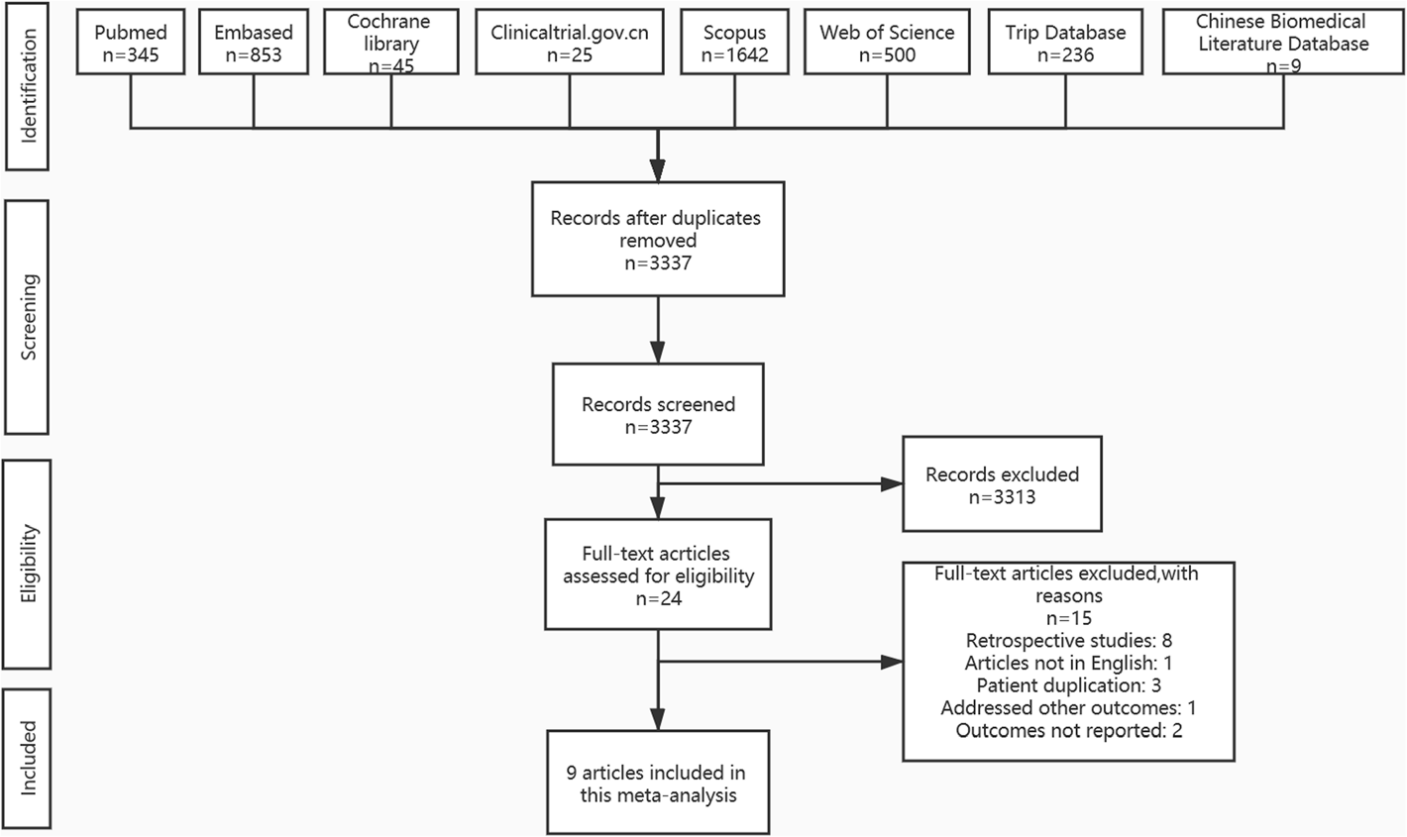
Flowchart of study selection

We identified 3655 articles in *PubMed, EMBASE, The Cochrane Library, ClinicalTrials.gov, Scopus, Web of Science, Chinese Biomedical Literature Database and Trip database.* Of these, 318 were excluded due to being duplicates. We selected 24 studies after title and abstract screening. Finally, nine studies [2, 22–29] were included in the meta-analysis after full text reading. Figure 1 shows the flowchart of study selection.

### Study characteristics

Among the nine studies included in this meta-analysis, two were RCT [22, 24], one was double-blind, placebo-controlled, crossover study [23], one was longitudinal comparative study [25], one was follow-up comparative study [26] and the remaining were non-comparative studies [2, 27–29].

Characteristics of all trials are shown in Table 1. Notably, the patients were treated with a high-dose first-generation SAs (octreotide or lanreotide) and PEG in all of the studies. All studies enrolled both male and female patients aged from 23 to 74 years old. Most of the patients received surgery as their initial therapy. Other previous treatments included radiotherapy and medical treatment (octreotide, lanreotide and cabergoline). The duration of the selected trials was also variable, ranging from 24 to 456 weeks.

**Table 1:** Characteristics of included trials

Author, year	Study Design	No. of Patients (Male/Female)	Age (y) Mean ± SD or Range	Baseline IGF-1 level (μg*L^−1^) Mean ± SD	Previous Treatment (n)	Mean or Median dosages of SAs and PEG	Outcomes	Duration of SAs and PEG treatment	Duration of Study/months
Jorgensen, 2005 [29]	Non-controlled, Prospective	11 (7/4)	23–71	269 ± 29	Surgery (9), Radiotherapy (5), SAs(10)	Octreotide: 30 mg/2–4 weeks PEG: 15 mg/day	IGF-1 levels, GH levels, FPG, FPI, 2-h plasma glucose value	Octreotide: not reported PEG: 12 weeks	8
Neggers, 2008 [23]	Placebo-controlled, Crossover, Prospective	20 (11/9)	39–74	187.5 ± 35	Both surgery and radiotherapy (6), Surgery (15), Primary medical therapy (5)	SAs: Not reported PEG: 40 mg/week	IGF-1 levels, glycosylated hemoglobin, FPG, TC, low-density lipoprotein, TELETs, Patient-Assessed Acromegaly Symptom Questionnaire, Acromegaly Quality of Life Questionnaire	SAs: 32 weeks PEG: 32 weeks	9
Trainer, 2009 [22]	RCT	84 (44/40)	24–70	626.477 ± 49	Surgery, Octreotide	Octreotide: 30 mg/28 days PEG: 15 mg/day	IGF-1 levels, acromegaly signs and symptoms, The Acromegaly Quality of Life Questionnaire, Euro Quality of Life-5 Dimensions Questionnaires, Glycaemic control, adverse event	Octreotide: 44 weeks PEG: 40 weeks	10
Madsen, 2011 [24]	RCT	18 (7/11)	54.2 ± 10.9	Not reported	Surgery (14), Radiotherapy (2), SAs (18)	Octreotide: 6.7-20 mg/28 days Lanreotide: 24-60 mg/28 days PEG: 52.5 mg/week	IGF-1 levels, GH levels, 2-h glucose, FPG, FPI, Insulin sensitivity and substrate metabolism, Quality of life, Adverse effects	SAs: 24 weeks PEG: 24 weeks	6
Van der Lely, 2011 [27]	Non-controlled, Prospective	57 (29/28)	51.6 ± 12.7	Not reported	Surgery (38), Radiotherapy (18), SAs (44), Lanreotide (24), Octreotide (20), PEG (13)	Lanreotide: 120 mg/months PEG: 60 mg/week	IGF-1 levels, acromegaly symptoms, Acromegaly Quality of Life Questionnaire, glucose tolerance, electrocardiograms, hepatic function tests, standard hematological, biochemical laboratory tests	Lanreotide: 44 weeks PEG: 28 weeks	7
Colao, 2019 [25]	Longitudinal, Comparative, Prospective	31 (14/17)	44.6 ± 10.5	643	SAs	Lanreotide: 40 mg/28 days PEG: 70 mg/week	Biochemical control rate, Acromegaly-related clinical signs and symptoms, Health-related quality of life, Safety	SAs: 8 months PEG: 4 months	8
Muhammad, 2018 [26]	Follow-up, Prospective	30	53	Not reported	Surgery, Radiotherapy, SAs	Pasireotide: 60 mg/28dyas PEG: 64 mg/week	Percentage of responders, Percentage of patients who stop PEG after 48 weeks, Percentage cumulative PEG dose reduction, Safety	Pasireotide: 36 weeks PEG: 48 weeks	12
Auriemma, 2016 [28]	Non-controlled, Prospective	36 (14/22)	52.3 ± 10.2	703.6 ± 258.3	Surgery, Radiotherapy, Octreotide (11), Lanreotide (25)	Octreotide: 30 mg/28 days Lanreotide: 120 mg/28 days PEG: 100 mg/week	IGF-1levels, cardiovascular parameters, glucose and lipid levels, tumor volume	SAs: 114 months PEG: 78 months	78
Urbani, 2013 [2]	Non-controlled, Prospective	31 (15/16)	46.47 ± 2.33	Not reported	SAs (31)	Octreotide: 27.50 mg/28 days Lanreotide: 120 mg/28 days PEG: 16.9 mg/day	IGF-1 index, GH, Glucose metabolism	SAs: not reported PEG: not reported	48

*RCT* randomized controlled trial, *SAs* somatostatin analogues, *IGF-1* insulin-like growth factor 1, *GH* growth hormone, *TC* total cholesterol, *TELETs* transient elevated liver enzyme tests

### Quality assessment of studies

The included studies had similar methodological quality and had no major defects in describing patient inclusion criteria, data collection and outcome evaluation (see the result of quality assessment in Additional file 2).

### Outcomes

#### IGF-1 normalization

**Fig. 2:**
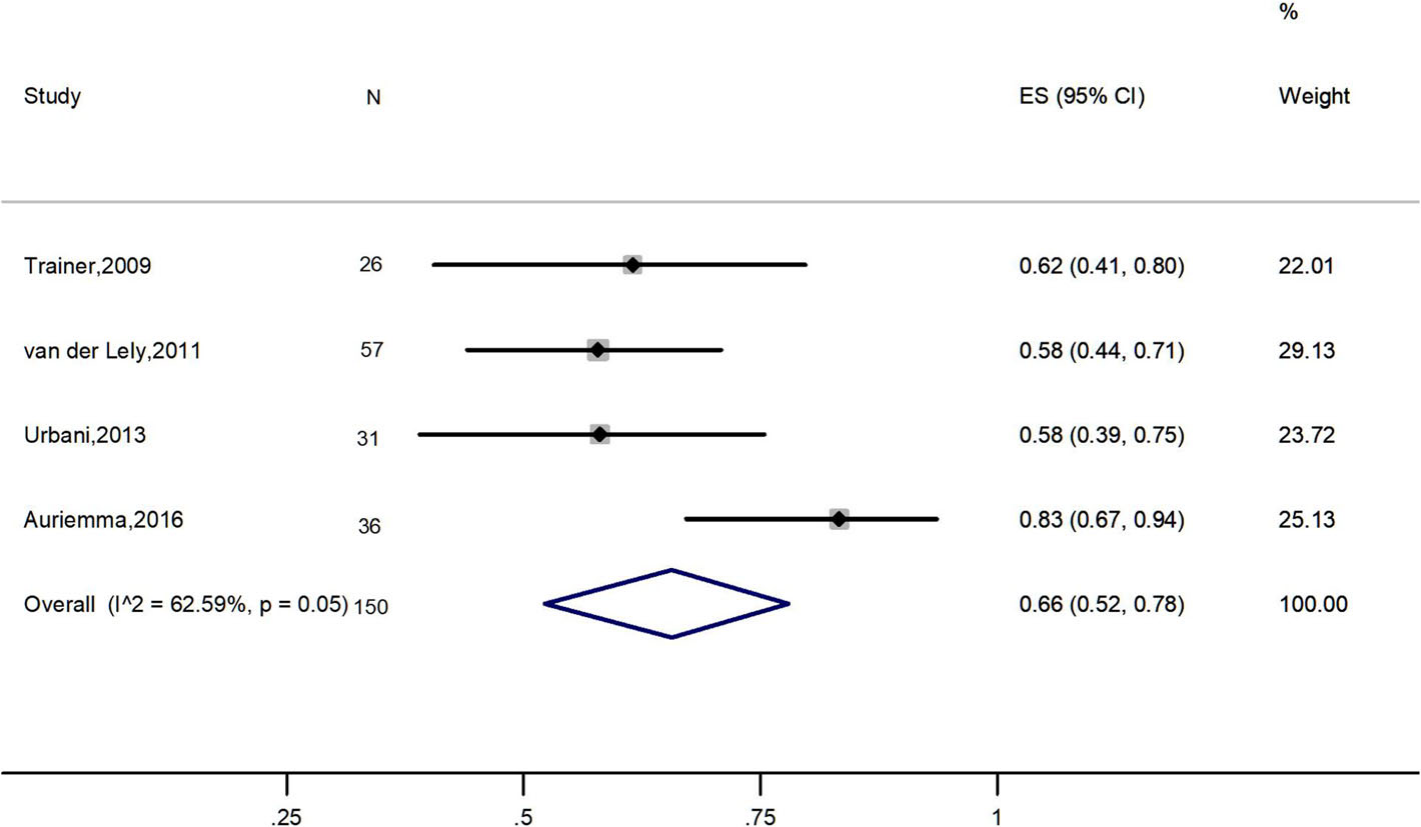
Meta-analysis results for the rate of patients achieving IGF-1 normalization

Four studies, 150 patients, were included in this analysis [2, 22, 27, 28]. In these studies, all patients had IGF-1 levels that were greater than the upper limit of normal (ULN) despite SAs monotherapy. Overall, the meta-analysis demonstrated an IGF-1 normalization rate of 66% (95% CI: 52–78%; Fig. 2), with high heterogeneity (*I*^2^ = 62.59%).

#### Glucose metabolism

**Fig. 3:**
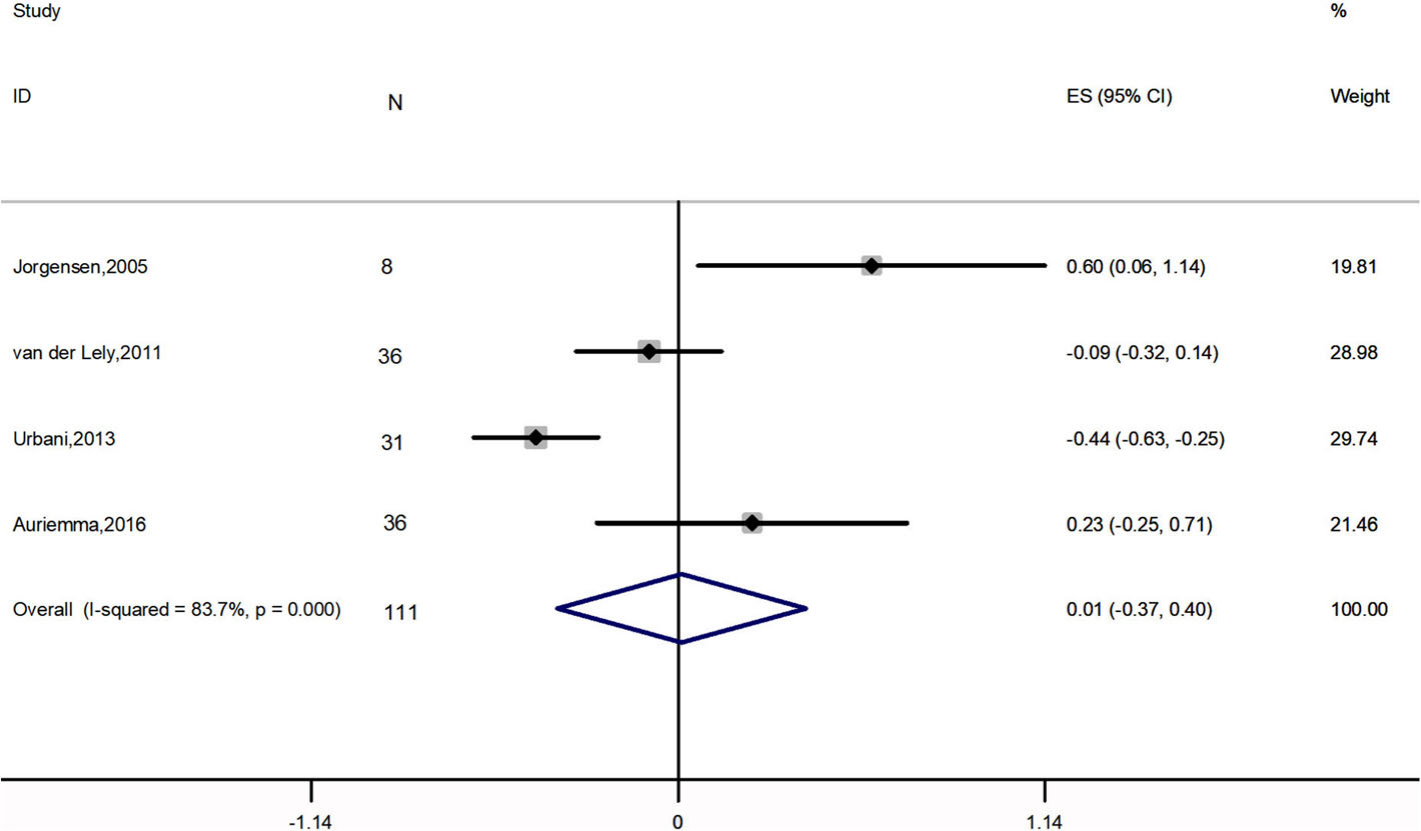
Meta-analysis results for the change in fasting plasma glucose

**Fig. 4:**
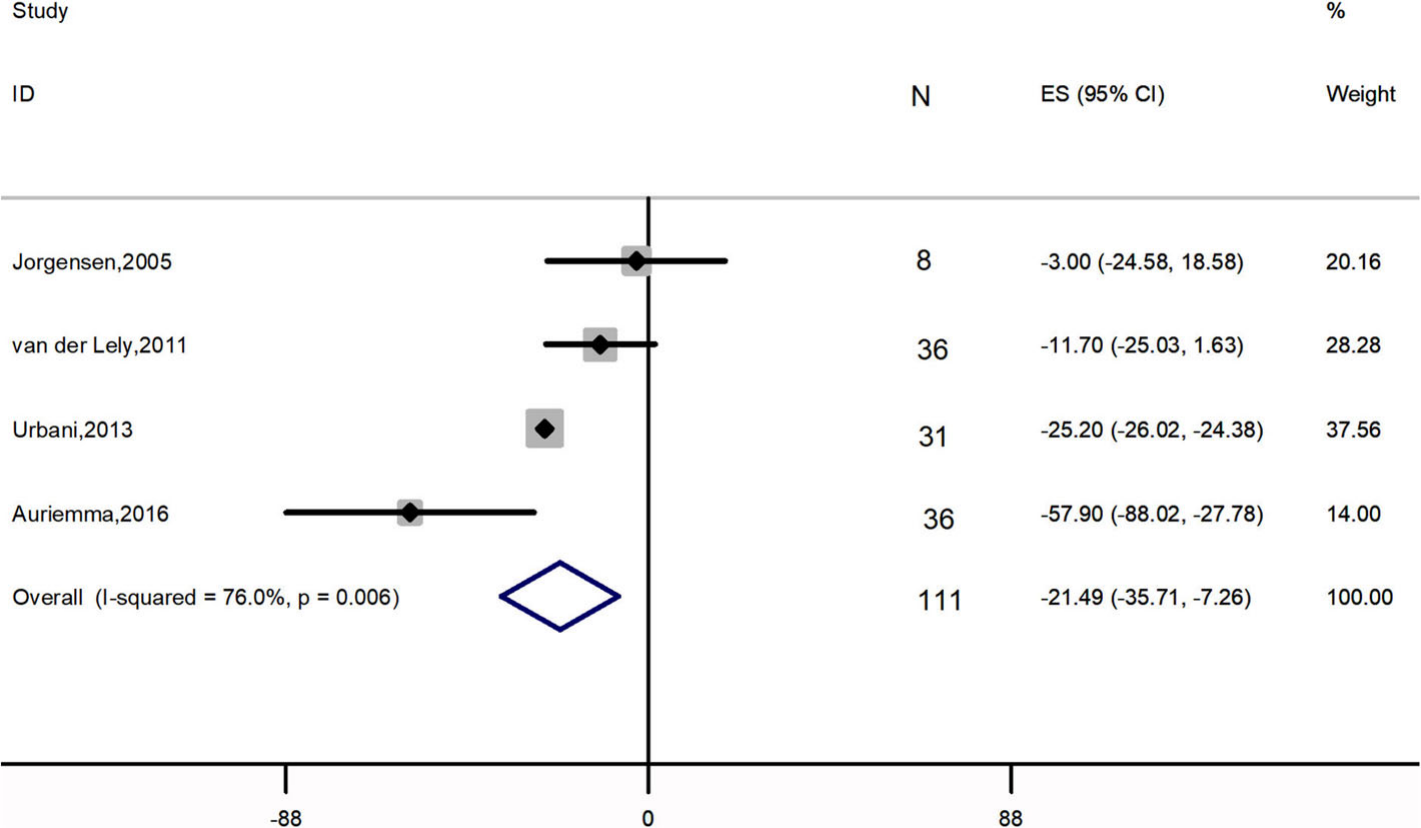
Meta-analysis results for the change in fasting plasma insulin

**Fig. 5:**
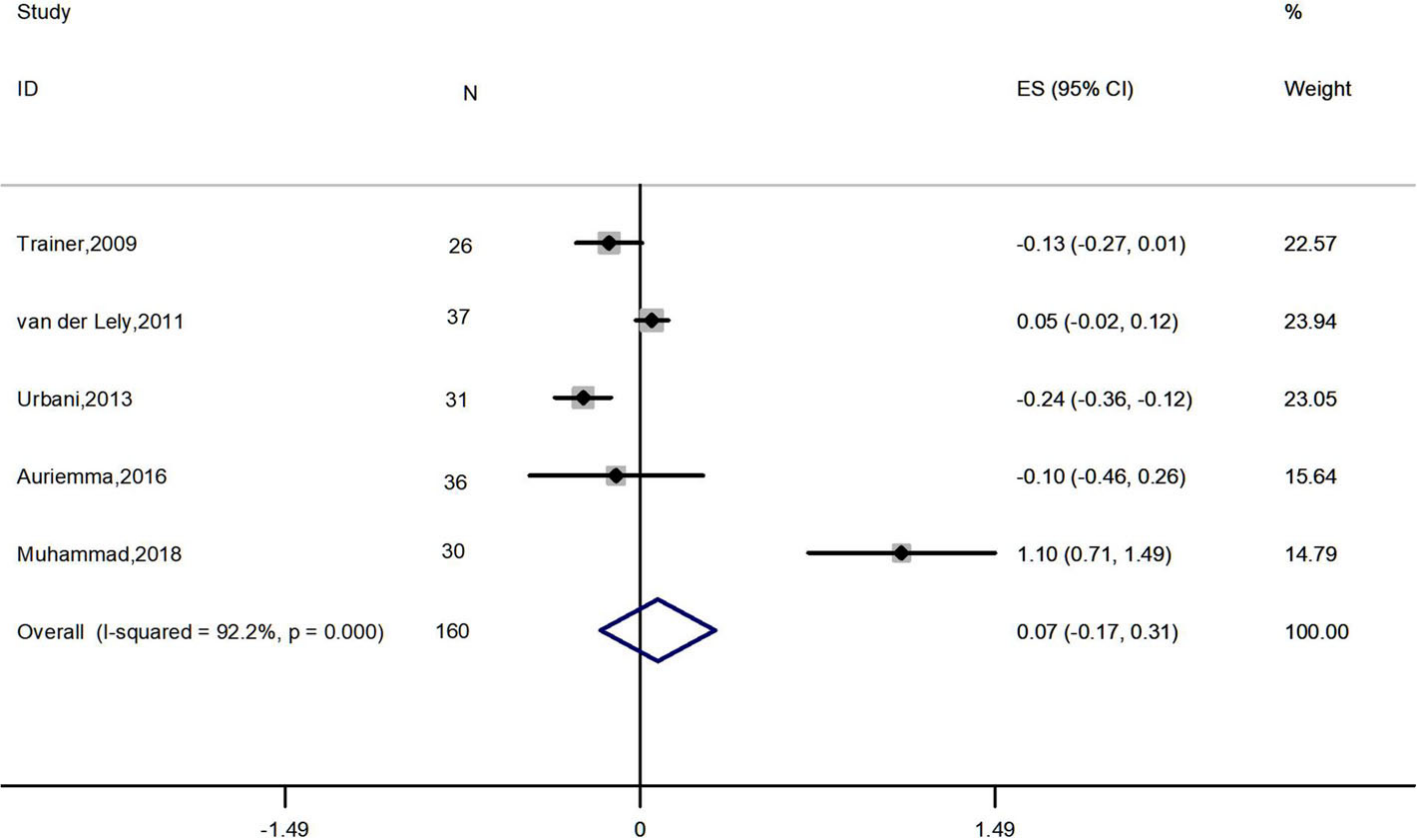
Meta-analysis results for the change in glycosylated hemoglobin

Meta-analysis of 4 studies, 111 patients, [2, 27–29] showed that the combination therapy did not significantly affect FPG (ES: 0.011 mmol*L^− 1^; 95% CI: − 0.374 to 0.397 mmol*L^− 1^; *I*^2^ = 83.7%; *P* = 0.954) (Fig. 3). Four studies [2, 27–29], 111 patients, were included in the analysis of FPI (ES: − 21.487 pmol*L^− 1^; 95% CI: − 35.713 to − 7.260 pmol*L^− 1^; *P* = 0.003), with high heterogeneity (*I*^2^ = 76.0%), revealing significant change (Fig. 4). Five papers, 160 patients, reported HbA1c levels before and after treatment [2, 22, 26–28]. A NOT significant decrease was found (ES: − 0.074%; 95% CI: − 0.166 to 0.315%; *P* = 0.544), accompanied by high heterogeneity (*I*^2^ = 92.2%) (Fig. 5).

#### Elevation in liver enzyme levels

**Fig. 6:**
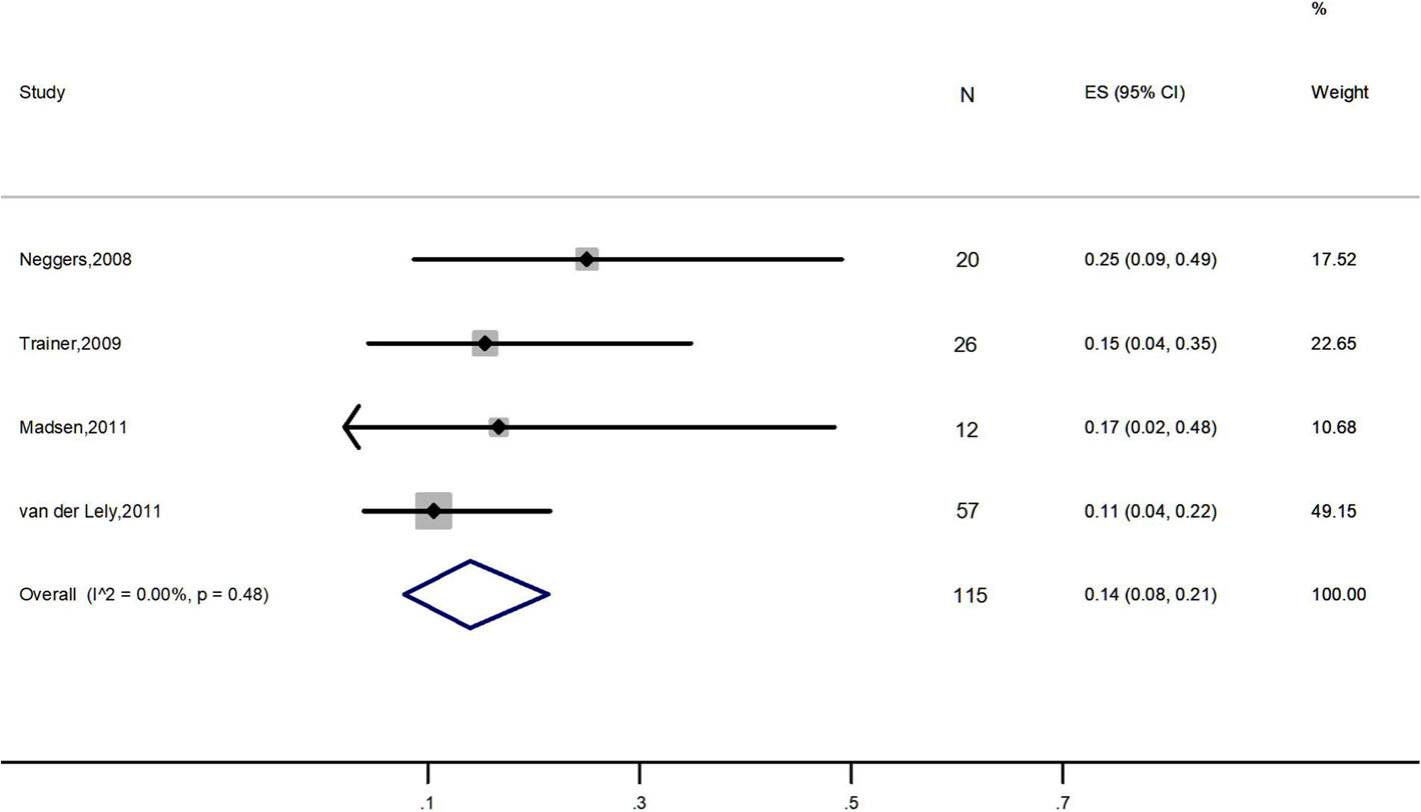
Meta-analysis result for the rate of patients with elevated liver enzyme levels

Four studies, 115 patients, were included in this analysis [22–24, 27]. Results demonstrated that the overall rate of elevation in liver enzyme levels was 14% (95% CI: 8 to 21%; *I*^2^ = 0%) (Fig. 6).

#### Other safety outcomes

We combined data for common safety outcomes reported in two comparative studies [22, 25], including serious adverse event and treatment discontinuation due to adverse event (Table 2). There was no significant difference between the combination therapy and SAs monotherapy. The evidence quality was low and moderate for serious adverse event and treatment discontinuation due to adverse event, respectively (see the result of evidence quality in Additional file 3).

**Table 2:** Summary of meta-analysis of safety outcomes

Outcome	Studies	Participants	Statistical method	Effect estimate	*P*-value	*I*^*2*^(%)	Evidence quality
Serious adverse event	2 [22,25]	92	Odd ratio (M-H, Fixed, 95% CI)	0.894 [0.102, 7.808]	0.919	0	Low
Treatment discontinuation due to adverse event	2 [22,25]	92	Odd ratio (M-H, Fixed, 95% CI)	4.288 [0.539, 34.141]	0.169	34.2	Moderate

#### Sensitivity analysis

Four meta-analyses showed high heterogeneity (Figs. 2, 3, 4 and 5). Thus, sensitivity analysis was performed to explore the heterogeneity. After removal of van der Lely’s and Muhammad’s studies [26, 27], a significant decrease of HbA1c levels was found (ES: − 0.189%; 95% Cl: − 0.276% to − 0.102%; *P* = 0.000), with no heterogeneity. For other outcomes, no significant differences were found compared with the initial analyses.

## Discussion

This meta-analysis gave us a more accurate understanding of the effect of PEG in association with SAs on acromegaly.

This meta-analysis revealed that combination therapy of SAs and PEG normalized IGF-1 in 66% of patients whose IGF-1 levels were still higher than ULN despite high-dose of SAs monotherapy, which was in accordance with Leonart’s study [15]. The meta-analysis showed high heterogeneity, the use of different assays for IGF-1 concentration and different PEG dose adjustment protocols may account for this variance.

Glucose metabolism impairment, from impaired glucose tolerance to severe diabetes mellitus, is frequently observed in acromegaly [30], which complicates the management of the disease. The prevalence of diabetes in acromegalic patients ranges from 20 to 56%, and that of impaired glucose tolerance ranges from 16 to 46% [3]. Over the past few decades, the effect of SAs on glucose homeostasis remains open [31, 32]. SAs exert antisecretory and antiproliferative effects by acting on somatostatin receptors subtype 2 (SSTR-2) and SSTR-5 [33]. SSTR-2 is mainly involved in glucagon regulation while SSTR-5 plays a role in regulating insulin secretion [34]. In addition, glucagon-like peptide-1, which plays an important role as an incretin hormone, is also modulated by SAs [35]. All of these pathways lead to the raise or lowering of blood glucose levels. Thus, it is difficult to confirm the net effect of SAs on glucose metabolism. Conversely, several studies have shown that PEG monotherapy induced a significant decrease in fasting glucose levels and HbA1c in patients with diagnosed diabetes mellitus or impaired glucose tolerance [2, 36–38]. A recent meta-analysis demonstrated that PEG induces a considerable decrease in FPG, HbA1c, FPI and in homeostatic model assessment of insulin resistance [18]. A positive effect of PEG on peripheral insulin sensitivity has also been demonstrated [39]. According to our results, the combination of SAs and PEG lead to a significant decrease in FPI which is in agreement with the previous meta-analysis [18]. It was expected since both drugs have the same effect on FPI. However, the combined treatment did not change the patients’ FPG or HbA1c levels, which is consistent with a previous study [18]. The reduced dose of PEG may be one of the reasons for this result. It is notable that a significant decrease of HbA1c level was found after the removal of two studies [26, 27]. Compared with other three studies [2, 22, 28], van der Lely’s study [27] had the shortest follow-up period and the glucose metabolism outcomes were evaluated only in non-diabetic patients. A one-year study also found that the combined treatment improved HbA1c level of acromegalic patients [40]. We, therefore, assume that long-term combined treatment may have a beneficial effect on HbA1c. Muhammad’s study demonstrated that HbA1c level increased from 6.0 to 7.1% after the combination therapy [25]. The patients in Muhammad’s study [25] were treated with pasireotide, a second-generation SAs, while patients in other four studies [2, 21, 26, 27] were treated with first-generation SAs, octreotide and lanreotide. A previous study showed that pasireotide may have worse effect on glucose metabolism compared with octreotide or lanreotide [41]. Overall, given the neutral global effect of PEG and SAs combination therapy on metabolic parameters, life style management and hypoglycemic drugs may be necessary in acromegalic patients who have impaired glucose tolerance or diabetes mellitus during PEG and SAs treatment.

Over the decades, SAs and PEG have been shown to be safe and well tolerated [42, 43]. Some surveillance studies [36, 44] reported an elevation of liver transaminase levels in approximately 5–8% of patients mainly previously treated with SAs. Transaminase level elevations during PEG treatment were often mild and transient [36]. The frequency of elevated liver enzymes seemed to increase in patients who were treated with a combination of SA and PEG [36]. In accordance with that, the present analysis of elevated liver enzymes showed an overall rate of 14%. The abnormal biochemical parameters returned to normal after treatment discontinuation in most of the patients. Nevertheless, we recommend careful monitoring of liver function during the cotreatment. Interestingly, a previous study with a small population [45] reported that acromegaly patients with diabetes mellitus had a 5.1 times higher risk than nondiabetic subjects for developing transiently elevated liver enzymes, but according to two papers [23, 27] included in this work, there was no relationship between diabetic status and elevated transaminases. Thus, more high-quality studies are needed to clarify their relationship.

The reduced dose of PEG is one of the advantages of this combination therapy. To maintain stable IGF-1 levels during PEG monotherapy, the mean weekly dose has been reported to be approximately 150 mg [44]. In four studies [2, 22, 27, 28] that were included in the analysis of IGF-1 normalization, the median effective weekly PEG doses were 119 mg, 60 mg, 80 mg and 105 mg. PEG is a much more expensive treatment than high-dose SAs treatment. Hence, we assume that combination therapy is less costly compared with PEG monotherapy.

Our meta-analysis has some limitations. First, most of the included papers are not RCTs. Second, the heterogeneity of some of the meta-analyses is high, and this was partially reduced by sensitivity analyses. Third, we could not get all of data that we were interested in because negative results were not reported and are likely to influence our results. Fourth, few of the analysed studies included patients after previous radiotherapy. The effect of PEG and SAs combination therapy may be partly due to the prolonged effects of radiotherapy on the pituitary function. Last, the number of studies included in the quantitative analyses is not large, but this situation is common when conducting a meta-analysis of a rare disease due to the lack of numerous original trials.

## Conclusion

This meta-analysis investigated the efficacy and safety of combined SAs with PEG therapy on acromegalic patients. Our analysis revealed that the coadministration of SAs and PEG is effective in normalizing IGF-1 levels in patients whose IGF-1 levels are higher than ULN despite high-dose SAs monotherapy. The therapy also decreased significantly FPI levels with a neutral effect on glucose parameters in acromegaly patients. In addition, the combination therapy was found to be safe, although liver function monitoring is still needed during treatment.

## Supplementary information

**Additional file 1: Table S1.** Full search strategy in database.

**Additional file 2: Table S2.** Assessment of study quality using Cochrane risk-of-bias algorithm for controlled trials. **Table S3.** Assessment of study quality using modified Methodological Index for Non-randomized Studies for non-comparative studies.

**Additional file 3: Table S4.** Assessment of evidence quality for two safety outcomes.

## Data Availability

The data analyzed during the current meta-analysis is available from the corresponding author on reasonable request.

## Abbreviations

GH: Growth hormone
IGF-1: Insulin-like growth factor 1
SAs: Somatostatin analogues
PEG: Pegvisomant
FPG: Fasting plasma glucose
FPI: Fasting plasma insulin
HbA1c: Glycosylated hemoglobin
CIs: Confidence intervals
ES: Effect size
ULN: Upper limit of normal
SSTR-2: Somatostatin receptors subtype 2
SSTR-5: Somatostatin receptors subtype 5
