# Supplementary material for: Combined therapy of somatostatin analogues with pegvisomant for the treatment of acromegaly: a meta-analysis of prospective studies
Source: BMC Endocr Disord. 2020 Aug 18;20:126. doi: 10.1186/s12902-020-0545-2 (PMC7433060; doi:10.1186/s12902-020-0545-2)
Supplement: Supplementary file 1 — Additional file 1: Table S1. Full search strategy in database. [file 12902_2020_545_MOESM1_ESM.docx]

**Additional Table 1. Full search strategy in database.**

| Database | Search strategy |
| --- | --- |
| PubMed | #1: ((Octreotide[MeSH Terms]) OR (Octreotide or Pasireotide or Sandostatin OR Somatuline OR lanreotide OR Somatostatin OR SSA OR SMS OR SRL)  #2: ((Acromegaly OR Acromegaly[MeSH Terms] OR Somatotropin Hypersecretion Syndrome OR Hypersecretion Syndrome, Somatotropin OR Inappropriate Growth Hormone Secretion Syndrome OR Inappropriate GH Secretion Syndrome))  #3: B2036-PEG OR Somavert OR Pegvisomant OR growth hormone receptor antagonist  #4: #1 AND #2 AND #3 |
| Embase | #1: acromegaly OR acromegalia OR acromegalism OR akromegalia OR megalakria  #2: pegvisomant OR 'b 2036' OR b2036 OR 'b2036 peg' OR 'pegylated somatotropin' OR somavert OR trovert OR 'growth hormone receptor antagonist'  #3: octreotide OR sandostatin OR somatuline OR lanreotide OR somatostatin OR ssa OR sms OR pasireotide  #4: #1 AND #2 AND #3 |
| Cochrane library | #1: 'Acromegaly OR 'Somatotropin Hypersecretion Syndrome' OR 'Hypersecretion Syndrome, Somatotropin' OR 'Inappropriate Growth Hormone Secretion Syndrome' OR 'Inappropriate GH Secretion Syndrome'  #2: Octreotide OR Sandostatin OR Somatuline OR lanreotide OR Somatostatin OR SSA OR SMS OR Pasireotide  #3: Pegvisomant OR growth hormone receptor antagonist  #4: #1 AND #2 AND #3 |
| Clinicaltrial.gov | Condition or disease: acromegaly  Other terms: pegvisomant |
| Scopus | #1:acromegaly  OR  acromegalia  OR  acromegalism  OR  akromegalia  OR  megalakria  OR  "Somatotropin Hypersecretion Syndrome"OR"Hypersecretion Syndrome, Somatotropin" OR "Inappropriate Growth Hormone Secretion Syndrome" OR "Inappropriate GH Secretion Syndrome"  #2: b2036-peg  OR  somavert  OR  pegvisomant  OR  "growth hormone receptor antagonist"  OR  "b 2036"  OR  b2036  OR  "b2036 peg"  OR  "pegylated somatotropin"  OR  somavert  OR  trovert  #3:octreotide  OR  sandostatin  OR  somatuline  OR  lanreotide  OR  somatostatin  OR  ssa  OR  sms  OR  pasireotide  #4: #1 AND #2 AND #3 |
| Web of Science | #1:acromegaly  OR  acromegalia  OR  acromegalism  OR  akromegalia  OR  megalakria  OR  "Somatotropin Hypersecretion Syndrome"OR"Hypersecretion Syndrome, Somatotropin" OR "Inappropriate Growth Hormone Secretion Syndrome" OR "Inappropriate GH Secretion Syndrome"  #2: b2036-peg  OR  somavert  OR  pegvisomant  OR  "growth hormone receptor antagonist"  OR  "b 2036"  OR  b2036  OR  "b2036 peg"  OR  "pegylated somatotropin"  OR  somavert  OR  trovert  #3:octreotide  OR  sandostatin  OR  somatuline  OR  lanreotide  OR  somatostatin  OR  ssa  OR  sms  OR  pasireotide  #4: #1 AND #2 AND #3 |
| Chinese Biomedical Literature Database* | #1: 生长抑素类似物 OR 奥曲肽 OR 兰瑞肽 OR 帕瑞肽  #2：肢端肥大症  #3：培维索孟 OR 生长激素受体拮抗剂  #4：#1 AND #2 AND #3 |
| Trip database | Population: acromegaly  OR  acromegalia  OR  acromegalism  OR  akromegalia  OR  megalakria  OR  "Somatotropin Hypersecretion Syndrome"OR"Hypersecretion Syndrome, Somatotropin" OR "Inappropriate Growth Hormone Secretion Syndrome" OR "Inappropriate GH Secretion Syndrome"  Intervention: b2036-peg  OR  somavert  OR  pegvisomant  OR  "growth hormone receptor antagonist"  OR  "b 2036"  OR  b2036  OR  "b2036 peg"  OR  "pegylated somatotropin"  OR  somavert  OR  trovert |

*Chinese local database
