# Supplementary material for: Combined therapy of somatostatin analogues with pegvisomant for the treatment of acromegaly: a meta-analysis of prospective studies
Source: BMC Endocr Disord. 2020 Aug 18;20:126. doi: 10.1186/s12902-020-0545-2 (PMC7433060; doi:10.1186/s12902-020-0545-2)
Supplement: Supplementary file 2 — Additional file 2: Table S2. Assessment of study quality using Cochrane risk-of-bias algorithm for controlled trials. Table S3. Assessment of study quality using modified Methodological Index for Non-randomized Studies for non-comparative studies. [file 12902_2020_545_MOESM2_ESM.docx]

**Additional Table 2. Assessment of study quality using Cochrane risk-of-bias algorithm for controlled trials.**

| Author, year | SELECTION BIAS | PERFORMANCE BIAS | DETECTION BIAS | ATTRITION BIAS | REPORTING BIAS | OTHER BIAS |
| --- | --- | --- | --- | --- | --- | --- |
| Neggers, 2008[23] | Unclear | Unclear | High risk | Low risk | Unclear | Unclear |
| Trainer, 2009[22] | Unclear | Unclear | High risk | Low risk | High risk | Unclear |
| Madsen, 2011[24] | Unclear | Unclear | High risk | Low risk | Low risk | Unclear |
| Colao, 2019[25] | Unclear | Unclear | High risk | Low risk | High risk | Unclear |
| Muhammad, 2018[26] | Unclear | Unclear | High risk | Low risk | High risk | Unclear |

**Additional Table 3. Assessment of study quality using modified Methodological Index for Non-randomized Studies for non-comparative studies.**

| Author, year | A clearly stated aim | Study population clearly defined | Prospective collection of data | Endpoints appropriate to the aim of the study | Unbiased assessment of the study endpoints | Follow-up period appropriate to the aim of the study | Loss to follow up less than 5% | Prospective calculation of the study size | Total Score |
| --- | --- | --- | --- | --- | --- | --- | --- | --- | --- |
| Jorgensen, 2005[29] | **2** | **1** | **2** | **2** | **0** | **2** | **1** | **1** | **11** |
| Van der Lely, 2011[27] | **2** | **2** | **2** | **2** | **0** | **2** | **2** | **2** | **14** |
| Auriemma, 2016[28] | **2** | **2** | **2** | **2** | **0** | **2** | **2** | **0** | **12** |
| Urbani, 2013[2] | **2** | **2** | **2** | **2** | **0** | **2** | **2** | **0** | **12** |

*The items are scored 0 (not reported), 1 (reported but inadequate) or 2 (reported and adequate).
