# Supplementary material for: Combined therapy of somatostatin analogues with pegvisomant for the treatment of acromegaly: a meta-analysis of prospective studies
Source: BMC Endocr Disord. 2020 Aug 18;20:126. doi: 10.1186/s12902-020-0545-2 (PMC7433060; doi:10.1186/s12902-020-0545-2)
Supplement: Supplementary file 3 — Additional file 3: Table S4. Assessment of evidence quality for two safety outcomes. [file 12902_2020_545_MOESM3_ESM.docx]

| Additional Table 4. Assessment of evidence quality for two safety outcomes | | | | | |
| --- | --- | --- | --- | --- | --- |
| Outcomes | Anticipated absolute effects^*^ (95% CI) | | Relative effect (95% CI) | № of participants  (studies) | Certainty of the evidence (GRADE) |
|  | Risk with [SSA monotherapy] | Risk with [The combination therapy] |  |  |  |
| serious adverse event (SAE) follow up: median 8 | 29 per 1,000 | 26 per 1,000 (3 to 230) | Rate ratio 0.894 (0.102 to 7.808) | 92 (2 RCTs) | ⨁⨁◯◯ LOW ^a,b^ |
| Treatment discontinuation due to adverse event follow up: median 8 | 0 per 1,000 | 0 per 1,000 (0 to 0) | OR 4.288 (0.539 to 34.141) | 92 (2 RCTs) | ⨁⨁⨁◯ MODERATE ^a,b^ |

#### CI: Confidence interval; OR: Odds ratio

#### Explanations

a. open label study

b. 1、small population；2、Low incident rate
